# The antibiotic susceptibility patterns of uropathogens among children with urinary tract infection in Shiraz

**DOI:** 10.1097/MD.0000000000007834

**Published:** 2017-09-15

**Authors:** Gholamreza Pouladfar, Mitra Basiratnia, Mojtaba Anvarinejad, Pejman Abbasi, Fatemeh Amirmoezi, Samaneh Zare

**Affiliations:** a Alborzi Clinical Microbiology Research Center, Nemazee Teaching Hospital; b Division of Pediatric Nephrology; c Hematology Research Center; d Student Research Committee, Shiraz University of Medical Sciences, Shiraz, Iran.

**Keywords:** antibiotics, pediatrics, Shiraz, urinary tract infection (UTI)

## Abstract

Urinary tract infection (UTI) is one of the most common bacterial infections in pediatrics. Delay in diagnosis and treatment can cause significant morbidity. The physician^'^s knowledge regarding the symptoms, microorganisms that caused UTI, and effective antibiotics in a geographical area can help them to select the appropriate antibiotics. This study was performed to determine the prevalence of bacteria that cause UTI and their susceptibility to common antibiotics as well as the common symptoms and associated factors in children of Shiraz, Southern Iran.

This cross sectional study was performed among 202 children with UTI, aged 2 months to 18 years old, between August and November 2014 in pediatric medical centers of Shiraz University of Medical Sciences. Urine samples were collected using urinary catheter or suprapubic in children < 2 years and mid-stream in children over 2 years, respectively. The type of micro-organisms causing UTI was determined and evaluation of antibiotic susceptibility for each organism was assayed by the Kirby Bauer method using antibiogram test. Patient's information was collected through checking the medical documents and interview with parents.

Our results showed that the frequency of UTI was significantly higher in girls (70.3%) than in boys. The most commonly discovered pathogens were *Escherichia coli* (*E coli*) (51.5%), followed by *Klebsiella* spp. (16.8%), and *Enterococcus* spp. (9.9%). Overall susceptibility test showed the highest resistance to ampicillin (81.2%) and cotrimoxazole (79.2%), and the highest sensitivity to imipenem (90.1%) and Gentamicin (65.3%). Gram negative and positive bacteria showed the highest antibiotic resistance to amoxicillin (83.8%) and clindamycin (100%), respectively. In addition, production of extended spectrum beta lactamase (ESBL) was 69.2% and 30.8% in *E coli* and *Kelebsiella* respectively.

The efficacy of third generation of the cephalosporins was reduced because of the high rate of production of ESBL and drug resistance. These results inform the physician as to which antibiotics are appropriate to prescribe for the patient, as well as urine culture reports and following the patient's clinical response so that high antimicrobial resistance is not developed at the community level.

## 1 Introduction

Urinary tract infection (UTI) is one of the most common infectious diseases diagnosed in outpatients, with a high rate of annual global incidence.^[1–3]^ Although UTI is the most common serious bacterial illness among febrile infants and young children, delay in diagnosis and treatment can cause significant morbidity, including renal scarring, hypertension, impaired renal function, and end-stage renal disease (ESRD).^[4–6]^ The incidence of UTI is more common among boys until the age of 12 months; however, the pooled prevalence rate of febrile UTI in females is about 3-fold of circumcised males and UTI occurrence increases among uncircumcised male infants.^[7,8]^*Escherichia coli (E coli)* is by far the most commonly isolated organism in pediatric UTI with prevalence ranging from 80% to 90%^[9]^ followed by others such as *Enterococcus* species (spp.), *Enterobacter* spp., *Pseudomonas aeruginosa*, *Kelbsiella pneumoniae*, *Proteus mirabilis*, and *Staphylococcus* spp.^[10,11]^

In our region, nitrofurantoin (80.9%), gentamycin (77.9%), and amikacin (65.3%) had the most in-vitro antibacterial effect on *E coli* isolates as the predominant cause of UTIs.^[12]^ The common practice in the empirical treatment of UTI in Iran is the prescription of nitrofurantoin or cefexime in afebrile children and gentamycin or cefexime in febrile ones.

The classic signs of UTI and pyelonephritis such as frequency, urgency, dysuria flank, and supra-pubic pain in older children and adults are not present or easily discerned in the toddler or young child. Fever is the most common nonspecific symptom of UTI in infants that might have different sources such as otitis media or other viral symptoms.^[13,14]^

Prevalence estimates can help clinicians make informed decisions regarding diagnostic testing and appropriate antibiotic therapy in children presenting with signs and symptoms of urinary tract infection.

Therefore, the aim of this study is to determine the prevalence of common bacteria causing UTI and their susceptibility to common antibiotics as well as the common symptoms and associated factors for UTI in children of Shiraz.

## 2 Patients and methods

In this cross-sectional prospective study, 276 patients at ages 2 months to 18 years, suspected of urinary tract infection, were evaluated in Shiraz University of Medical Sciences, Southern Iran between July and November 2014, and finally 202 patients with confirmed UTI were recorded. Twenty-two patients were excluded because of negative culture. Patients who were referred to hospital pediatric emergency room were considered outpatients, and patients who were admitted to the ward or intensive care unit (ICU) and developed UTI at or after 3 days of admission, were considered inpatients with nosocomial infections. This study was approved by the medical ethics committee and consent form was completed before starting the study.

Inclusion criteria were: children 2 months to 18 years of age presenting with symptoms of UTI or only fever with suspected UTI source in infants, a positive urine culture of a single organism according to standard guidelines. Patients who have received antibiotics before urine culture or samples that grew more than 1 type of micro-organism or fungal infection were excluded from the study.

Urine samples were collected suprapubicly or by transurethral catheterization from patients younger than 2 years old and using the Midstream Specimen of Urine in patients with 2 years of age or older in sterile containers. A UTI was defined as ≥10^5^ colony-forming units (CFU)*/*mL of midstream urine, ≥10^4^ CFU*/*mL of urine obtained by transurethral catheterization, and ≥10^3^ CFU/mL of gram-positive microorganism or any colony number of gram-negative micro-organism by suprapubic collection.

Samples were cultured on blood agar or MacConkey agar by using a standard calibrated loop (0.01 mL) and the plates were incubated at 37°C for 24 hours. After identifying the bacteria, the antibiotic susceptibility test was performed by the Kirby–Bauer disk diffusion based on Clinical and Laboratory Standards Institute 2013.^[15]^ Susceptibility of gram-negative bacteria was tested against disks of nitrofurantoin, nalidixic acid, ampicillin, colistin, cefotaxime, ceftazidime, piperacillin- tazobactam, imipenem, piperacillin, ciprofloxacin, chloramphenicol, ceftriaxone, aztreonam, gentamicin, amikacin, ampicillin- sulbactam, cotrimoxazol, tetracycline, tobramycin, cephalexin, amoxicilin, cefixime, meropenem, cefepime, and ticarcilin. Disks used for gram-positive bacteria included: nitrofurantoin, nalidixic acid, ampicillin, cefotaxime, imipenem, ciprofloxacin, chloramphenicol, ceftriaxone, aztreonam, gentamicin, trimethoprim/sulfamethoxazole (TMP-SMX), tetracycline, cefixime, cefepime, vancomycin, linezolid, cefoxitin, cloxacillin, penicillin G, rifampin, erythromycin, azithromycin, and clindamaycin.

The spread of extended spectrum beta lactamase (ESBL) among *E coli* and *Kelebsiella* species was screened according to Clinical and Laboratory Standards Institute 2011 guidelines using confirmatory disk diffusion methods.^[16]^ A cefotaxime (30 μg) and a cefotaxime + clavulanic acid (30+ 10 μg), ceftazidime (30 μg) and ceftazidime + clavulanic acid (30+ 10 μg) discs (Mast, Basingstoke, UK) were placed at a distance of 25 mm on a Mueller-Hinton Agar plate, inoculated with a bacterial suspension of 0.5 McFarland turbidity standards and incubated overnight at 37°C. A ≥ 5 mm increase in the diameter of inhibition zone for the combination disc versus ceftazidime disc, confirmed ESBL production. ESBL producing strain *Kelebsiella pneumoneae* ATCC 700603 and non-ESBL producing strain *E coli* ATCC 25922 were used as positive and negative controls.^[17]^

Data collected included age, gender, hospital status (inpatient or outpatient), urine collecting route, clinical findings, antibiotic consumption during the last 3 months, and lab data.

After data collection, SPSS version 18 was used for the statistical analysis. Descriptive data were presented as mean, standard deviation, median, and range. The *χ*^2^ test was used for comparison of qualitative variable among different groups. *P* value less than .05 was considered statistically significant.

## 3 Results

A total of 202 urinary specimens of different patients with UTI were included in the study. The mean age of the patients was 5.34 ± 5.87 years including 60 boys (29.7%) and 142 girls (70.3%). 43.6% of patients were younger than 2 years of age. More than half of the patients (56.4%) were admitted to the hospital. Sampling was done using catheter in 50.5%, midstream in 43.6%, and suprapubic in 5.9% of children. About half of the cases (46.5%) had fever, and dysuria, frequency, abdominal pain, vomiting, malodor urine, anorexia, and failure to thrive were present in 32%, 8.9%, 26.7%, 16.8%, 17.8%, 37.6%, and 6.9% respectively. According to findings on sonography the patients who had hydronephrosis and hydroureter were about 2-fold of the patients with bladder hypertrophy (14.9% vs 8.9%). Positive history of antibiotics usage during last 3 months was the most prevalent associated factor for UTI in our study (49.5%). Other associated factors that made the patients prone to urinary tract infection were history of previous UTI, constipation, intermittent catheter insemination (ICI), immunosuppressive drugs usage, vesicoureteral reflux, and urinary tract stones, respectively. Based on our data, fever as a major symptom, and history of previous UTI and ICI as associated factors, had statistically strong relation with micro-organism types (*P* value < .05). Table 1 details the findings in different groups of micro-organisms.

**Table 1:**
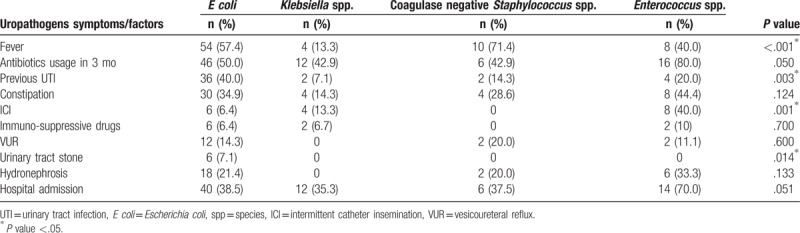
The prevalence of UTI symptoms and associated factors among different groups of uropathogens.

The most commonly discovered pathogens were *E coli* (51.5%; 95% CI = 44.4%–58.6%), followed by *Klebsiella* spp. (16.8%; 95% CI = 11.9%–22.7%), and *Enterococcus* spp. (9.9%; 95% CI = 6.2%–14.9%). Coagulase negative *Staphylococcus* spp. (7.9%; 95% CI = 4.6%–12.5%), *Enterobacter* spp., *Pseudomonas* spp., *Streptococcus* spp., *Acinetobacter* spp., *Proteus* spp., and *Serratia marcescens* were responsible for about 22% of uropathogens. Altogether, there were twice as many girls with UTI than boys (*P* value = .003). The prevalence of UTI in girls with *E coli*, *Enterococcus* spp., and Coagulase negative *Staphylococcus* spp. uropathogens was statistically higher than boys (*P* value < .05). The prevalence of the 4 most common microorganisms isolated from outpatients was higher than that from inpatients, except for *Enterococci* spp. Moreover, *Pseudomonas* spp. was the cause of UTI only in inpatients.

**Figure 1:**
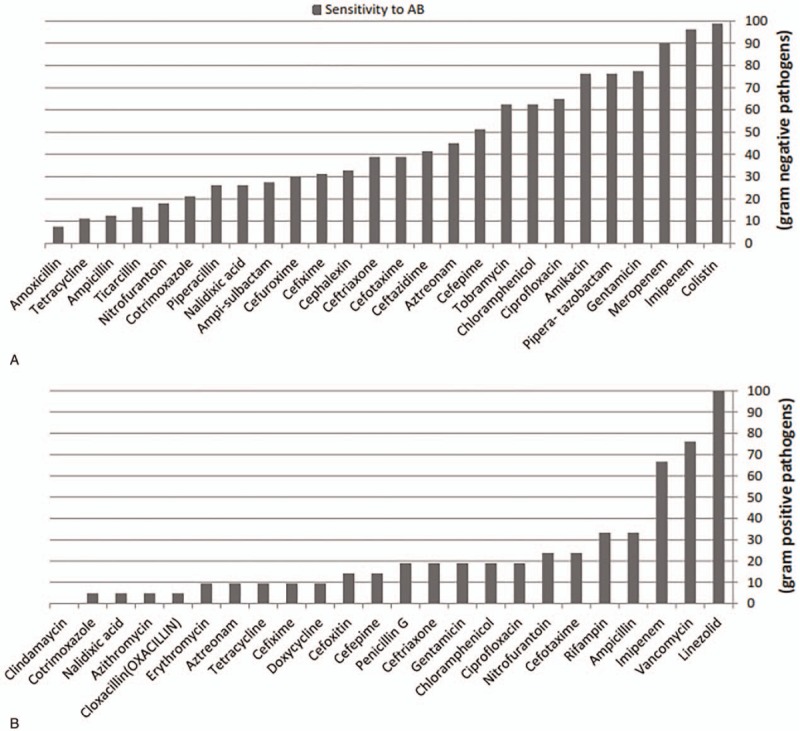
Sensitivity test of 160 gram-negative (A) and 42 gram-positive (B) microorganisms. AB = antibiotic.

Overall susceptibility test showed the highest resistance to ampicillin (82.3%) and TMP-SMX (77.3%), and the highest sensitivity to imipenem (93.4%) and gentamicin (65.3%). Table 2 shows antimicrobial resistance patterns of isolated uropathogens in detail. According to our results, the most effective antimicrobial agents for gram-negative bacilli were colistin (98.8%) and imipenem (96.2%) as intravenous (IV), gentamicin (77.5%), and amikacin (76.3%) as intramuscular (IM) or IV and ciprofloxacin (55.4%) as oral usage. The appropriate IV antimicrobial agents for gram-positive cocci were linezolid (100%) and vancomycin (76.2%), and linozolid as oral usage (Fig. 1). According to our study, 40% of isolated *Enterococcus* spp. were vancomycin resistant *Entrococci*. Moreover, 69% of *E coli* (n = 104) and 50% of *Klebsiella* spp. (n = 32) had ESBL.

**Table 2:**
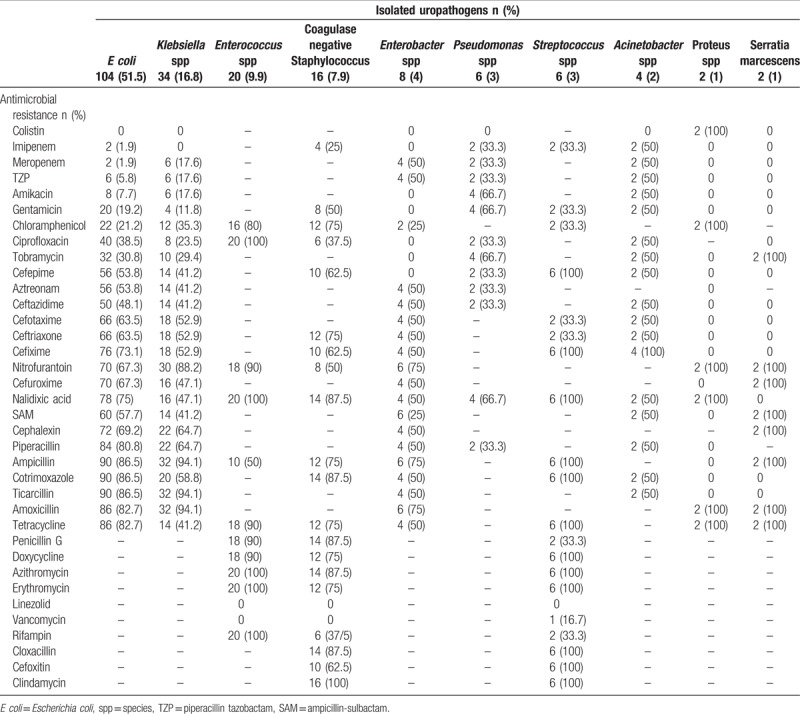
Antibiotic resistance patterns of isolated uropathogens.

## 4 Discussion

The results of our study showed that *E coli* was generally found to be the most frequent cause of UTI in children in Shiraz, Southern Iran with the mean prevalence of 50.5% and there was a trend to reduce third-generation cephalosporin sensitivity of uropathogens. Similar frequency of isolates of *E coli* has been obtained in studies performed in different regions of Iran with maximum distribution of 78.1% in the west.^[18–23]^ Similar to many other studies, we found a higher rate of UTI among female children.^[23–26]^ Girls older than 2 years generally have an increased association of UTI due to anatomic shortness of urethra and close space between anus and urethral orifice.

However, there were almost equal numbers of positive cases in both male and female during infancy.^[24]^ A meta-analysis in 2008 emphasized the important role of circumcision status in determining the association for a UTI and implied that uncircumcised male infants less than 3 months of age had the highest prevalence of UTI whereas circumcised males had one of the lowest rates.^[7]^

Hospital acquired urinary tract infection is a common infection especially in ICU admitted patients. It was almost always associated with the use of an invasive device like urinary catheter.^[27–29]^ Diabetic and immune-deficient patients are more susceptible to hospital acquired UTI.^[30]^ On the basis of our results, the most common organisms isolated from inpatients were *E coli* (45.4%). These findings are compatible with other studies in which *E coli* was reported as the most common uropathogen in nosocomial UTIs (21%).^[27,29,31,32]^

Ilić et al^[33]^ in Croatia showed that *E coli* was the most frequent isolate (67.7%) with resistance to ampicillin by 69.5%, amoxicillin/clavulonic acid by 3.5%, cephalexin by 6.6%, TMP-SMX by 27.5%, and nitrofurantoin by 0.4%. Another study on 25,418 American outpatients with UTI revealed that resistance among *E coli* was highest for TMP-SMX (24%) but lowest for nitrofurantoin (less than 1%) and cephalothin (15%).^[34]^ Some studies in our region determined the susceptibility of *E coli* to different antimicrobial agents; in a study from the center of Iran susceptibility of *E coli* to nitrofurantoin was 85.7%, gentamicin 82.5%, cefotaxime 81.9%, and ciprofloxacin 78% and high resistance rate was observed against ampicillin (100%).^[22]^ In western Iran high susceptibility patterns to: ciprofloxacin (95.3%), amikacin (93.9%), nalidixic acid (92.2%), gentamicin (89.2%), and nitrofurantoin (83.8%) among the *E coli* isolates identified were observed.^[20]^ High resistance to cephalothin and ceftriaxone and high susceptibility to nitrofurantoin, ciprofloxacin, and nalidixic acid in the north were seen.^[18,35]^ Literature from Kerman, in the east of Iran, revealed that almost all uropathogenic *E coli* were sensitive to carbapenems (100%) and amikacin (94.4%), while 100% and 63.8% of the strains were resistant to ampicillin and amoxicillin/clavulanic acid, respectively.^[21]^

Our study is in agreement with some studies that introduced *Klebsiella* spp. as the second most common uropathogen^[12,20,21,25,36]^ which displays a similar resistance pattern as for *E coli*. Based on the findings in the present study, for gram-negative bacilli the highest resistance to amoxicillin and highest sensitivity to colistin, imipenem, and gentamicin and in gram-positive cocci the highest resistance to clindamycin and TMP-SMX and highest sensitivity to linezolid and vancomycin were seen. High rate of vancomycin resistant *Entrococci* was detected in this study which was reported up to 71.4% in health-care acquired UTI from our center.

Although third-generation cephalosporins were indicated in UTI in the recent past, the resistance against them increased at the time of our study. It can be due to the high level of ESBL that some organisms produced. The present study was in agreement with our previous article that reported about 61% of *Klebsiella pneumonia* and 35% of *E coli* isolates were ESBL producing in our region.^[17]^ Over-prescribing of oral third-generation antibiotics such as cefexime, which is a common practice in Iran, increases drug resistance among gram-negative pathogens, especially through producing ESBL (i.e., antibiotic selection pressure).^[31]^

Intramuscular gentamicin and oral ciprofloxacin were the best candidates in outpatient treatment; however, trends toward increased resistance to these antibiotics were seen.

Our study faced some limitations. First, results should be re-evaluated in larger studies. Therefore, generalization of the study result to all of the patients with UTI is not free from bias. Furthermore, a potential bias is that our patients were selected from a referral and tertiary hospital.

These results make the physicians aware of which antibiotics are appropriate to be prescribed, accompanied with urine culture reports and following the patient's clinical response so that high antimicrobial resistance is not developed at the community level.

## Acknowledgments

The authors thank all persons at the Professor Alborzi Clinical Microbiology Research Center, Shiraz, IR Iran for their technical assistance.
